# The Molecular Biology and Replication Cycle of Infectious Pancreatic Necrosis Virus

**DOI:** 10.3390/v18040436

**Published:** 2026-04-03

**Authors:** Daniela Espinoza, Jorge Gómez, Ana María Sandino, Sebastián Gonzalez-Catrilelbún, Andrea Rivas-Aravena

**Affiliations:** 1 Laboratorio de Virología, Escuela de Bioquímica, Universidad San Sebastián, Santiago 7510157, Chile; 2 Laboratorio de Virología, Centro de Biotecnología Acuícola, Universidad de Santiago de Chile, Santiago 9170022, Chile; ana.sandino@usach.cl; 3 Departamento de Ciencias Biológicas y Químicas, Facultad de Ciencias, Universidad San Sebastián, Santiago 7510157, Chile

**Keywords:** IPNV, birnavirus, RNA virus, IBDV, RNA replication strategy

## Abstract

*Infectious pancreatic necrosis virus* (IPNV), a member of the family *Birnaviridae*, is a major pathogen of farmed salmonids and an important model in fish virology. Despite its small genome, which encodes only five viral proteins, IPNV exhibits complex molecular processes that govern genome expression, replication, and particle assembly. Comprehensive descriptions of the molecular biology and replication cycle of IPNV were largely established in reviews published in the mid-1990s, whereas more recent reviews have primarily focused on virulence determinants, epidemiology, or host–virus interactions. This review provides an updated synthesis of available experimental knowledge on the molecular biology of IPNV by integrating classical and recent studies addressing virion architecture, genome organization, and the functions of viral proteins. Particular attention is given to the molecular events involved in the viral replication cycle, including virus entry, genome transcription, translation and replication in the cytoplasm, polyprotein processing by the viral protease, and the coordination between genome replication and virion assembly. When appropriate, experimental observations from the related *Avibirnavirus* infectious bursal disease virus are considered to provide additional context for molecular mechanisms conserved within the family *Birnaviridae*. Together, these studies outline the current understanding of the molecular processes governing IPNV replication and morphogenesis.

## 1. Introduction

The family *Birnaviridae* comprises seven genera: *Aquabirnavirus* (infecting fish; including *Infectious Pancreatic Necrosis Virus*, IPNV; *Yellowtail ascites virus*, YAV; and *Tellina virus 2*, TV-2), *Avibirnavirus* (infecting birds; *Infectious Bursal Disease Virus*, IBDV), *Blosnavirus* (infecting fish; *Blotched snakehead virus* and *Lates calcarifer birnavirus*), *Entomobirnavirus* (infecting insects; *Drosophila X virus*, DXV, and *Mosquito X virus*, MoXV), *Dronavirus* (infecting insects; *Drosophila-B birnavirus*), *Ronavirus* (infecting rotifers; *Rotifer birnavirus*, RBV), and *Telnavirus* (infecting molluscs; *Tellina virus 1*, TV-1) [1]. Additionally, two Birnavirus strains have been recently characterized: the *Paralichthys olivaceus birnavirus*, POBV, classified within the genus *Aquabirnavirus*, and the *Mandarin fish birnavirus*, MFBV [2,3].

Among birnaviruses, the prototypical and most studied members are IPNV and IBDV, due to their considerable economic impact on aquaculture and poultry farming, respectively.

Birnaviruses exhibit similarities and differences in their genomic, structural, and replicative features relative to other single-stranded RNA (ssRNA) and double-stranded RNA (dsRNA) viruses [4,5,6,7]. Their genome comprises two segments of dsRNA (segments A and B) packaged within a single, unenveloped icosahedral capsid [8,9]. Similar to +RNA viruses, each genomic segment is covalently linked to a VPg protein at the 5′ end [10,11,12] and translated as a polyprotein precursor [13]. Unlike canonical dsRNA viruses that rely on rigid transcriptional cores, birnaviruses package ribonucleoprotein (RNP) complexes consisting of dsRNA, the RNA-dependent RNA polymerase (RdRp, VP1), and VP3 [14,15,16]. In IBDV, these RNPs are required for RNA protection [17,18] and for transcriptional functionality of the viral genome, retaining transcriptional activity outside the capsid when reconstituted in vitro [19,20]. Altogether, these features illustrate that birnaviruses could blend molecular strategies of +RNA and dsRNA viruses, foreshadowing their role as a possible evolutionary bridge within the RNA virosphere [18,19,20].

The single-capsid, T = 13 icosahedral architecture of birnaviruses and the jelly-roll fold structure of the capsid protein closely resemble those of several non-enveloped +RNA viruses, such as nodaviruses and tetraviruses, suggesting a shared structural ancestry despite the dsRNA genome [4,5,6,21]. Additionally, the birnavirus RdRp VP1 exhibits a permuted polymerase motif arrangement—the canonical A-B-C motif order is rearranged to C-A-B—an uncommon trait also observed in certain +RNA viruses [7,22,23].

Additionally, the packing of the birnaviral genome does not follow the common features of dsRNA virus encapsidation, where the viral genome is never exposed to the cellular environment, being synthesized inside the viral core [24]. Instead, replication occurs in the cytoplasm [9,19,25,26].

Among them, IPNV, recently renamed *Aquabirnavirus Salmonidae* [27], is one of the most extensively studied members, both as a model for birnavirus molecular biology and due to its considerable economic impact on aquaculture. IPNV causes a high-prevalence disease in intensively farmed Atlantic salmon (*Salmo salar*), rainbow trout (*Oncorhynchus mykiss*), and coho salmon (*Oncorhynchus kisutch*), although it can also infect other salmonids and non-salmonid fish. The virus is responsible for recurrent outbreaks that lead to significant mortality in juvenile fish and substantial financial losses in salmonid aquaculture worldwide [28,29]. In surviving fish, the infection can become persistent, resulting in lifelong carriers that serve as reservoirs for viral transmission [30].

Due to its economic relevance, several vaccines have been developed with varying degrees of success in disease prevention. Importantly, for almost 15 years, IPNV-resistant salmon have been farmed in several producing countries [31]. These strains carry a quantitative trait locus (QTL) conferring resistance to infection. However, the rapid evolution of IPNV has led to the emergence of new virulent variants, causing outbreaks even in resistant stocks [32,33,34], highlighting the virus’s adaptability.

While several reviews have focused on IPNV pathogenesis in infected salmon [35,36,37,38,39,40,41], this review explores its replicative cycle and molecular biology, highlighting parallels with IBDV in areas where experimental evidence for IPNV is still lacking. However, such comparisons need to be incorporated cautiously: although both viruses belong to the family *Birnaviridae* and share a bisegmented dsRNA genome organization, they differ markedly in biology and host context. IBDV infects avian hosts and specifically targets immature B lymphocytes in the bursa of Fabricius, leading to lymphoid depletion and immunosuppression in young chickens [42]. In contrast, IPNV infects salmonids, causing infectious pancreatic necrosis predominantly at lower water temperatures (∼10–18 °C) and with primary target tissues including the pancreas, anterior kidney, and liver [43]. Moreover, the amino acid sequence identity between key proteins of IPNV and IBDV is relatively low. For example, homologous viral polymerases show only about 40% identity [44,45], limiting the extent to which molecular mechanisms can be reliably extrapolated between the two systems.

## 2. IPNV Biochemical Characteristics

### 2.1. Virion Structure

IPNV is a non-enveloped virus with an icosahedral capsid of an average of 60 nm in diameter. The capsid is composed of the protein VP2, which encloses two RNP complexes, each corresponding to one of the dsRNA genome segments (A and B). The 5′ end of each RNA strand is covalently linked to a molecule of VP1, referred to as VPg (for genome-linked viral protein) [10,11].

IPNV was initially described as a heterogeneous population of viral particles ranging from 57 to 74 nm in diameter [46,47,48]. More recent analyses of particles recovered from the supernatant of fully lysed infected cells revealed populations that migrate differentially in CsCl gradients [49,50,51], displaying an even broader size distribution from 20–25 nm up to 90 nm in diameter. The 25 nm particles correspond to self-assembled VP2 structures released during ultracentrifugation of virions. The protein and genome content of these particles varies according to size: most contain all viral proteins except VP5, and particle diameter correlates with the number of genome segments encapsidated. Remarkably, IPNV can package 2, 3 (two copies of segment A and one of segment B), 4, or even 6 segments [49,51], a phenomenon also documented in IBDV [18].

### 2.2. Genome Organization and Viral Proteins

Based on VP2 phylogeny, IPNV isolates cluster into six genogroups that align with classical serotypes and their type strains. Genogroup 1 includes the American serotypes A1 (West Buxton, WB; VR-299; Buhl; Dry Mills, DM; DRT) and A9 (Jasper, Ja) [52,53]. The close relationship of DM and WB as A1 references is further supported by VP2/VP4 sequence comparisons in wild North Atlantic fish isolates [54]. Genogroup 2 corresponds to A3 (Abild, Ab), Genogroup 3 to A5 (Tellina, Te) and A6 (Canada-1, C1), and Genogroup 4 to the Canadian serotypes A7 (Canada-2, C2) and A8 (Canada-3, C3) [53]. Genogroup 5 corresponds to the European A2 (Spajarup; Sp) [52,53], and Genogroup 6 to the singleton A4 (Hecht; He) [53].

Segment A (~3.1 kb) contains two open reading frames (ORFs): (i) the large ORF (ORF L), encoding the VP2–VP4–VP3 polyprotein, and (ii) the small ORF (ORF S), encoding VP5 in the +1 reading frame. ORF L is translated as a polyprotein: 5′–preVP2–VP4–VP3–3′, which is cleaved by the protease VP4 into the pre-VP2 (pVP2, ~60 kDa), which is consecutively processed to obtain VP2 (~55 kDa), VP4 (29 kDa), and the RNA-binding protein VP3 (31 kDa) [13,55,56,57].

VP2 is the most abundant viral component and derives from the N-terminal region of the precursor pVP2. Early biochemical analyses by Dobos estimated approximately 550 VP2 molecules per virion [58,59], whereas more recent cryo-electron microscopy data suggest about 780 molecules per virion [60]. Both VP2 and pVP2 have been detected in purified virions [9,49]. VP2/pVP2 assembles into a single-protein icosahedral shell with T = 13 L symmetry (l for leavo) [61,62] and constitutes the major immunogenic component of the virus [63,64,65].

Although early lectin/[^3^H]-mannose labeling suggested that VP2 is glycosylated in virions [66], a later systematic study reported no detectable glycosylation in IPNV produced in CHSE-214 cells [67]. However, later work demonstrated that VP2 carries O-linked sugars added in the cytoplasm rather than the ER/Golgi [68,69]. Functionally, the modification appears modest in quantity but highly relevant in effect: removal of O-linked sugars by chemical oxidation leads to a dramatic loss of infectivity and destabilization of virions, while attachment to host cells remains unaffected. These findings suggest that O-GlcNAc on VP2 plays a structural rather than receptor-binding role, stabilizing intersubunit interactions within the capsid and protecting virions from premature disassembly [70].

IPNV provides an unusual example of a non-enveloped virus exploiting cytoplasmic glycosylation pathways to modify its structural proteins.

VP3 is an internal structural viral protein. It is the second most abundant protein in the virion (~544 molecules in the virion) [58,59]. Using a yeast two-hybrid system, it was shown that VP3 interacts with VP1, with itself, and with dsRNA in a sequence-independent manner [16]. The binding of hundreds of VP3 molecules to the genomic dsRNA forms the RNP complex that is ultimately packaged into the virion [15,61].

VP4 is the viral protease responsible for processing the birnaviral polyprotein. It cleaves the precursor at the (Ser/Thr)-X-Ala/(Ser/Ala)-Gly motif through a serine-protease mechanism that relies on a GxS nucleophilic motif and a Ser/Lys catalytic dyad, with Ser acting as the nucleophile and Lys as the general base [71,72,73]. Mutational and structural analyses established that this activity defines a novel class of viral serine proteases, mechanistically related to bacterial leader peptidases rather than classical chymotrypsin-like proteases [72]. Early studies estimated approximately 122 VP4 molecules per virion [58,59]. Interestingly, birnaviral proteases from different genera share low sequence homology with one another, typically less than 20% [72].

Truncated forms of VP4 have been reported in IPNV. Early evidence identified two shorter species of the nonstructural protein, termed NSt and NSta, detected both in infected cells and in purified virions by Western blotting, immunoprecipitation, and peptide mapping [9,56]. This self-cleavage activity can be abolished by introducing a mutation in the VP4 active site (Lys674 → Ala) [72]. Biochemical and structural analyses further confirmed that both provirions and mature virions contain full-length VP4 together with its truncated form [9]. However, the biological role of these truncated VP4 species remains unresolved.

The ORF S on segment A, located in the +1 reading frame relative to the polyprotein ORF, encodes the nonstructural protein VP5 [74]. In addition to the ORF L initiation codons (iAUG), segment A contains two in-frame AUGs located approximately at nucleotides 68 and 113, either of which could potentially serve as the start codon for VP5 translation. Experimentally, VP5 has been detected in multiple isoforms ranging from ~12 kDa (e.g., Sp, G5) to ~17 kDa (e.g., Jasper and N1, G1) [75,76,77,78]. In silico analyses of Chilean isolates predict additional VP5 length heterogeneity, including very short (~3.1 kDa) ORF Ss in some G1/G5 isolates and an extended ~29.6 kDa variant restricted to certain G5 isolates, although these forms have not yet been verified at the protein level [53]. Moreover, some highly virulent isolates harbor an in-frame premature stop codon (e.g., Sp, G5), yielding truncated VP5 isoforms of ~12 kDa [79] or ~15 kDa [80], whereas low-virulence isolates often contain an early stop codon that predicts a very short predicted VP5 of ~3.3 kDa [81].

VP5 has been reported to exert anti-apoptotic activity [74,76,82]; however, its role in infection remains controversial [77]. Regarding innate immunity, VP5 (together with VP4) can antagonize type I interferon pathways by inhibiting IFN-induced Mx promoter activity and dampening activation of the Atlantic salmon IFNa1 promoter in transfected cells [83].

Segment B (~2.9 kb) encodes VP1 (~94 kDa), a non-canonical RdRp that exhibits a permuted arrangement of the catalytic motifs characteristic of birnavirus [7,22,23]. Unlike most viral RdRps, birnavirus VP1 lacks the canonical Gly-Asp-Asp (GDD) motif [23,44], placing it within a distinct subgroup of RNA polymerases. In vitro assays demonstrated that VP1 possesses reverse transcriptase activity and template-independent self-guanylylation, with an N-terminal serine residue essential for RNA polymerase complex formation [84]. The genomic dsRNA contains a covalently linked 5′ VPg moiety corresponding to VP1 [10,12]. Additionally, VP1 also occurs as a free protein within the virion [10,12,69]. Quantitative estimations indicate that each virion contains approximately 22 VP1 molecules [58,59].

IPNV 5′ untranslated regions (5′UTRs) are short, conserved elements with clear functional relevance. The segment A 5′UTR is ~120 nt and the segment B 5′UTR is ~100 nt [57,75,85]. Both segment A and segment B 5′ UTRs contain two upstream AUGs, a feature commonly associated with internal ribosome entry sites (IRES). In segment A, one of these AUGs serves as the initiation codon for VP5 translation. Comparative analyses of 5′ UTRs across strains (e.g., West Buxton, Jasper, N1) show high nucleotide conservation and shared features such as short inverted repeats [75,85]. Furthermore, the alignment of the 5′-terminal sequences of IPNV-Jasper and N1 segments A and B revealed conservation of 32 out of 50 nucleotides [44,75]. Functionally, the segment A 5′UTR contains a bona fide IRES that drives translation in fish cells, whose secondary structure is sensitive to temperature, a property that likely modulates translation efficiency within the physiological thermal range of salmonid hosts [85,86].

The 3′UTRs are relatively short, approximately 80 nucleotides for segment A and 110 nucleotides for segment B and show a high degree of sequence conservation among isolates. These regions lack a poly(A) tail. Alignment analysis showed that 29 out of 50 nucleotides at the 3′ terminus of segment B were conserved in the segment A sequence [44,75]. Inverted repeat regions are also present in the 3′UTR. Mutational analyses and reverse genetics experiments revealed that the 3′ UTR of IBDV forms stem–loop motifs that are functionally relevant for viral infectivity, since mutations that disturb this secondary structure negatively impact viral infectivity, and the virus restitutes the secondary structure after only one passage concomitantly with the restitution of infectivity [87]. The 3′UTR functionality has not yet been investigated in IPNV.

## 3. The Viral Replication Cycle of IPNV

**Figure 1:**
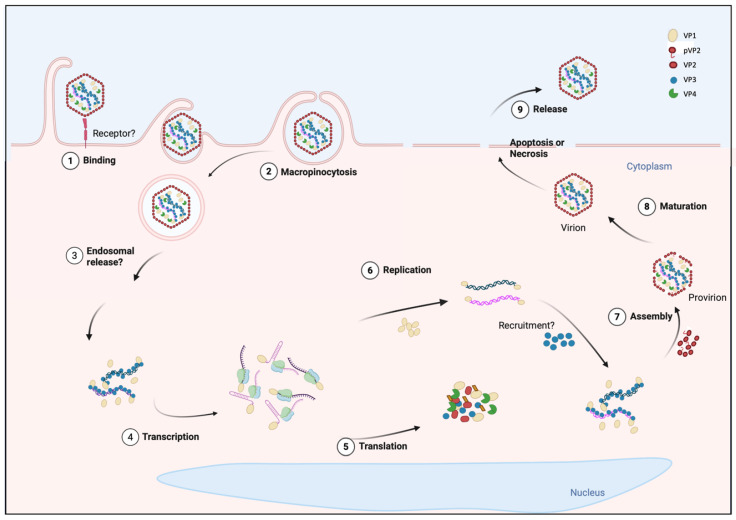
Schematic representation of the IPNV replication cycle. The process begins with viral binding to a putative receptor (1) and internalization by macropinocytosis (2), followed by endosomal release by an undetermined process (3). In the cytoplasm, VP1 initiates transcription from the RNP (4), followed by mRNA translation (5), leading to genome replication (6), assembly of RNPs (7), capsid maturation (8), and release of infectious virions (9). Steps inferred from experimental data are shown, and uncertain events are indicated with question marks. Created in BioRender. Rivas, A. (2026). https://BioRender.com/0bkrr5e.

Although the main stages of the IPNV cycle have been described through biochemical and microscopic analyses, their molecular coordination remains only partially understood. The entire process lasts approximately 16 to 24 h and comprises viral entry within the first 20–30 min; VPg-dependent transcription between 2 and 4 h post-infection (hpi); translation of viral proteins detected from 4 to 8 hpi; genome replication occurring between 8 and 12 hpi, with packaging taking place concomitantly with RNA synthesis; and the release of mature virions typically observed from 16 hpi onward [9,26,88,89] (Figure 1). This regulated cycle integrates replication strategies from both positive-sense and double-stranded RNA viruses and appears to involve subtle mechanistic variations depending on the viral strain.

### 3.1. Entry

The mechanism of IPNV entry into host cells is not fully understood, and several aspects of its entry process remain unclear. Before internalization, the virus requires binding to the cell surface (Figure 1, step 1). IPNV initially binds to specific, saturable receptor-mediated sites and to non-specific, non-saturable attachment sites; only the specific sites are functional, and these have been estimated at approximately 1500 receptors per cell, which mediate productive infection [90]. In this context, different studies have proposed different proteins as putative receptors for IPNV; however, no consensus has yet been reached. Initially, a membrane protein of approximately 220 kDa was identified in Atlantic Salmon Kidney (ASK), CHSE-214, an embryonic epithelial line from *Oncorhynchus tshawytscha*, and SHK-1 cells, a macrophage-like line derived from the head kidney of *Salmo salar*, as a potential receptor, but its identity was not determined [91]. In a later study, epithelial cadherin (Cdh1-1), detected as ~100 kDa fragments, was suggested to act as a receptor or co-receptor of the virus in Atlantic Salmon. This hypothesis originated from a QTL analysis that identified Cdh1-1 as the main determinant of resistance and was supported by co-immunoprecipitation assays showing its interaction with IPNV in liver tissue [92]. Later, non-muscle myosin heavy chain 9 (Myh9, 260 kDa) was identified as a critical factor for viral entry through its interaction with VP2 in CHSE-214 cells. However, no co-localization between E-cadherin and VP2 was observed by immunofluorescence, suggesting that the involvement of these molecules may vary depending on the cell type or may correspond to complementary functions at different stages of infection [93].

Following attachment, IPNV is internalized into peripheral vesicular compartments within the first 20–30 min after adsorption [90,94]. Available studies suggest that IPNV may predominantly enter host cells via macropinocytosis (Figure 1, step 2). This hypothesis is supported by experiments performed in CHSE-214 and SHK-1 cells. In both models, typical features of macropinocytosis were observed, including dynamic actin rearrangements, dose-dependent uptake of extracellular fluid, and co-localization with dextran in large intracellular vacuoles. In addition, infection was reduced by inhibitors of actin polymerization or the Na^+^/H^+^ exchanger, while remaining unaffected by classical blockers of clathrin-, caveolin-, or dynamin-mediated endocytosis [95,96]. Although these results strongly support macropinocytosis as the predominant entry mechanism, alternative endocytic pathways cannot be excluded. IPNV may exploit distinct internalization routes depending on the host cell type, such as clathrin-mediated internalization described in hepatocytes of *Salmo salar* [92].

Following macropinocytic uptake, the post-internalization steps that allow IPNV to access the cytosol remain unresolved. Acidification was initially proposed as a requirement for entry, since compounds such as ammonium chloride inhibited infection [97]. Nevertheless, later studies using more specific inhibitors of the vesicular H^+^-ATPase, such as bafilomycin A1, demonstrated that IPNV viral cycle progression does not depend on endosomal acidification, and that the effects of ammonium chloride previously observed could be attributed to cellular toxicity [98].

In contrast, although IBDV is internalized through macropinocytosis as IPNV (83), its release strictly depends on endosomal acidification for entry. Its uncoating requires acidification of early endosomes, which regulates endosomal Ca^2+^ release and activates the pep46 peptide, derived from VP2 maturation, that deforms membranes and generates pores allowing RNP release [99,100,101]. The difference in dependence on acidification between IBDV and IPNV raises questions about its escape mechanism, specifically how it crosses the endosomal membrane despite the absence of endosomal acidification, a process that in many non-enveloped viruses triggers conformational rearrangements required for membrane penetration. In IPNV, VP2 has been shown to conserve membrane-perforating peptide sequences analogous to those of IBDV [99]. If these peptides were involved in viral release, their expression and activation would likely depend on alternative stimuli, such as receptor interactions (Cdh1-1 or Myh9) or changes in the vesicular microenvironment, that could promote conformational transitions exposing hydrophobic regions capable of inserting into membranes and forming pores [101]. The elucidation of the mechanism of IPNV entry into the cell could provide key insights into the molecular biology of the virus and guide the development of preventive antiviral strategies.

### 3.2. Transcription

**Figure 2:**
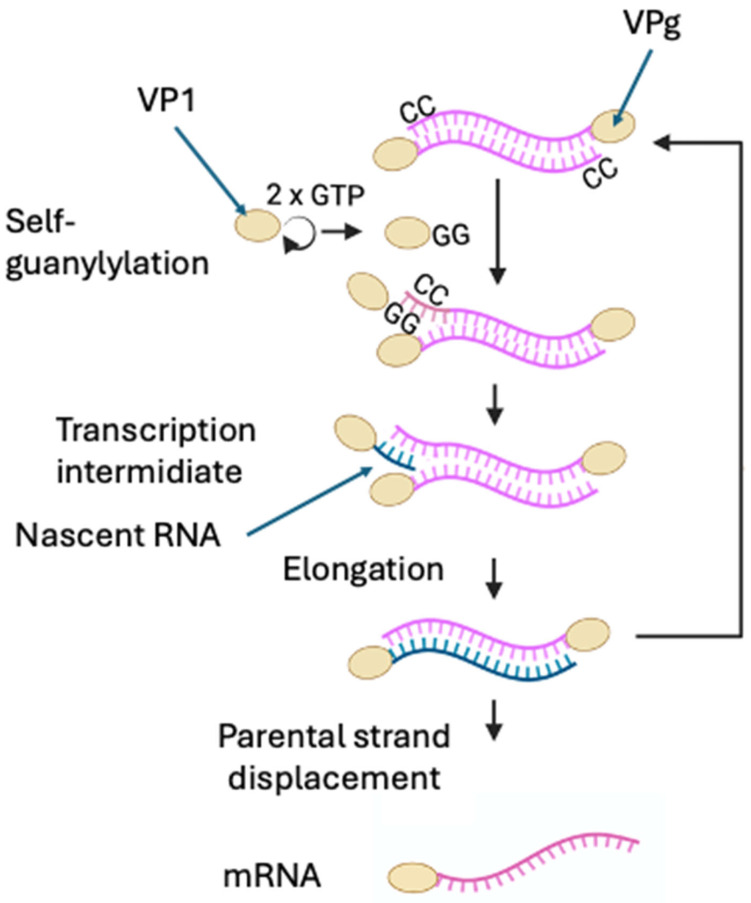
During transcription, VP1 undergoes two sequential self-guanylation reactions, generating the VPg form that remains covalently linked to the 5′ end of viral transcripts. VPg aligns as a primer by base pairing with the two conserved cytidine residues at the 3′ terminus of the RNA template. Primer extension is then carried out by RNA-dependent RNA polymerase activity, which may be provided either by a VP1 molecule acting in trans or by the same VPg-linked VP1 acting in cis; however, the exact mechanism remains unresolved. This process results in the synthesis of VPg-linked viral mRNAs from both genome segments. Created in BioRender. Rivas, A. (2026). https://BioRender.com/0bkrr5e.

Although significant progress has been made in elucidating the molecular mechanisms of IPNV transcription, several key aspects remain unresolved. IPNV mRNA synthesis initiates by a protein-primed, semi-conservative mechanism [11,102]. The self-guanylylation at a serine residue [102] maps to the N terminus, but the exact site is unclear: although Ser163 was initially proposed [103], this result was not reproduced in subsequent experiments [84]. Guanylylated VP1 aligns with cytidines at the 3′ end of the template strand, acting as a primer and producing RNA covalently linked to VPg at the 5′ end [10]. Therefore, IPNV mRNAs lack both a 5′ cap and a 3′ poly(A) tail, two modifications typically required for efficient translation and stability of eukaryotic mRNAs (Figure 2).

Once IPNV enters the host cell, the genomic dsRNA is proposed to be released into the cytoplasm as an RNP complex [15], since the genome remains in a VP3 RNP (Figure 1, step 3). This raises the question of whether transcription proceeds entirely within the RNP or requires partial disassembly of the RNP complex. Purified virions are transcriptionally active in vitro, indicating that VP1 is active prior to uncoating [11,104,105]. However, few VP1 molecules are packaged per virion [59], raising the question of how the virus maintains sufficient polymerase activity, given that free VP1 molecules are sequestered at 5′ ends in each RNA synthesis cycle. This uncertainty extends to whether transcription can be initiated and sustained by VPg within the RNP complex, or whether additional free VP1 molecules must be recruited from a cellular pool of newly synthesized VP1 during infection. Alternatively, it cannot be excluded that VPg itself retains the catalytic activity, supporting mRNA synthesis independently of free VP1, a question that remains experimentally unresolved [104,105]. Supporting this notion, studies on IBDV transcription have demonstrated that purified RNPs are transcriptionally active in vitro, strongly indicating that VPg-linked protein is catalytically competent within the RNP complex [106].

Classical nucleotide-incorporation assays showed that VP1 produces three viral RNA species in infected CHSE-214 cells [89]. The first RNA species (24S) was detected at 2–4 hpi and corresponded to the mRNA. A second form of RNA was detected between 6 and 12 hpi, corresponding to the dsRNA. A third form of viral RNA, composed of ssRNA and dsRNA with low electrophoretic mobility in 2% acrylamide-agarose gels, was called transcription intermediate (TRI), because it resembled a replicative intermediate but was detected as early as the mRNA. Supporting this, Magyar et al. (1998) showed in IPNV-infected cells, through immunoprecipitations and pulse-chase assays, that VP1 is linked to oligoribonucleotides of various lengths of +RNA [107]. Subsequent studies demonstrated protein-primed synthesis in which VP1 covalently attaches, as Vpg, to the genome via a serine–GMP linkage [10,11,89], establishing VP1 as both polymerase and primer, producing a Vpg-linked RNA [102] (Figure 1, step 4). Biochemical data indicate that VP1 carries out both activities: RNA chain elongation and protein-primed guanylylation [10,102,103], yet no structural evidence supports a distinct second catalytic site for GMP transfer in IPNV. By contrast, in IBDV, deletion of the polymerase active site does not abolish self-guanylylation, indicating that the polymerase and self-guanylylation activities are mechanistically separable [108]. Functional assays show that guanylylation at Ser163 is template-independent and chemically distinct from canonical RNA synthesis, suggesting mechanistic separation within the same enzyme while leaving the precise spatial organization unresolved [102,103].

In a more recent study, replication intermediates in infected cells were analyzed, and observed accumulation of both ss and partial dsRNA forms. In this study, the populations of RNP complexes were analyzed by buoyant CsCl gradients. Two peaks of labeled material were observed: 1.33 g/cm^3^, concordant with the viral particles, and 1.4 g/cm^3^, corresponding to different RNA species associated with viral proteins. The RNP population of 1.33 g/cm^3^ was associated with the genomic dsRNA from 7 hpi, and the RNP of 1.4 g/cm^3^ presented a heterologous population of both ssRNA and dsRNA [26]. Northern blot experiments performed to determine the nature of RNA generated during infection in CHSE-214 cells showed that full-length +RNA was detected at 4 hpi, consistent with the time of appearance of viral mRNA [89]. These results are consistent with the semi-conservative strand displacement mechanism [102], where the parental displaced strand serves as mRNA.

Purified IPNV virions and isolated VP1 are transcriptionally active in vitro in the absence of additional viral proteins [14,84,89,104]. In contrast, for IBDV, although VP1 expressed in insect cell lines is sufficient to generate transcripts from a cDNA template [109], subsequent studies showed that RNA synthesis within RNPs requires the structural protein VP3 [106], and that VP3 enhances VP1 activity to replicate a synthetic RNA containing the IBDV 3′ UTR [20]. Additionally, recent work has shown that IBDV replication components (VP1, VP3, and dsRNA) concentrate on PI3P-positive early-endosomal membranes, where a basic polypeptide patch in the C-terminal region of VP3 (K411, K412, K420, R423) mediates PI3P binding. This membrane platform nucleates viral replication/transcription condensates from which newly synthesized RNAs emerge into the cytoplasm [110].

The transcription strategy proposed for birnaviruses differs markedly from that of all other dsRNA viruses described to date, which rely on transcription within a core particle and the extrusion of mRNA outside the core [111]. For example, in reoviruses, double-layered particles synthesize capped mRNAs inside the particle: the polymerase (VP1) synthesizes RNA and the guanylyltransferase (VP3) caps nascent transcripts [24]. In reovirus, fivefold-axis pores serve as mRNA-exit and NTP-entry sites [112]. Interestingly, IBDV high-resolution structures define the T = 13 capsid and vertex depressions, but open fivefold pores functioning as RNA/NTP channels have not been demonstrated [5,113]. Furthermore, in IPNV, earlier cryo-EM reconstructions revealed the capsid lattice but no functionally defined channels. However, a recent 2.75 Å cryo-EM structure [61] showed a fivefold pore formed by five VP2 subunits at each vertex, occluded by flexible loops. An electropositive ring, created by VP2 Lys159 and Arg425, lines the pore interior, potentially attracting NTPs and supporting intraparticle genome synthesis by analogy to other dsRNA viruses. The pore radius is slightly larger in T = 13 pentons (~19.8 Å) than in T = 1 (~18.9 Å); penton views appear more open than trimer views, consistent with a gated architecture. The interior RNP remained unresolved, and functional gating or transcript egress has not yet been demonstrated, leaving the role of the pore in transcription inferential. The discovery of this structure in the axis of the IPNV capsid raises the question of whether IPNV transcripts exit through the fivefold pores, as observed in reovirus, or whether there is an alternative mechanism that decouples transcription from particle structures. Alternatively, the RNP could be released into the cytoplasm and interact with endosomal membranes. The precise cellular localization of transcription remains open. If IPNV mirrors IBDV’s membrane-associated factories in PI3P-positive early endosomes [110], such condensates could concentrate VP1, VP3, and genomic dsRNA to promote efficient synthesis while spatially segregating replication from host sensors (e.g., RIG-I, MDA5, PKR), thereby reducing dsRNA exposure [114].

### 3.3. Translation

Infection by IPNV in fish-derived cell lines causes a marked inhibition of host cellular mRNA translation. This translational shut-off is temporally associated with increased phosphorylation of eIF2α, a modification that has been attributed both to activation of PKR (protein kinase R) [115,116,117] and to PERK (protein endoplasmic reticulum kinase) [118]. PERK activation has been associated with endoplasmic reticulum (ER) stress and the engagement of the unfolded protein response (UPR) during IPNV infection [117,118].

VP1 appears to contribute to the cellular translation inhibition, as its expression either in vitro or in cells strongly suppresses cap-dependent translation, most likely through the hijacking of eIF4E, while allowing non-canonical translation [119]. Collectively, these findings support a model in which IPNV simultaneously engages UPR signaling and PKR/PERK-mediated eIF2α phosphorylation, together with VP1-driven eIF4E hijacking, to inhibit host cap-dependent translation. Under these conditions, IPNV mRNAs are preferentially translated via non-canonical recruitment of the initiation machinery, allowing continued viral protein synthesis despite global inhibition of host translation [87,119].

IPNV mRNAs, which are covalently linked to VPg at their 5′ ends and lack a poly(A) tail, encode one of the largest VPg proteins described among RNA viruses [119]. The size of VPgs in other viral families is much smaller: 22 amino acids (aa) in *Picornavirus* [120]; 28 aa in *Enamovirus* [121]; 78–79 aa in *Sobemovirus* [122], ~13–15 kDa in *Astrovirus* [123]; and ~21 kDa in *Calicivirus* [124] and *Potyvirus* [125]. The implications of this unusually large VPg for the regulation of viral transcription and translation remain largely unknown.

Functionally, the VPg linked to IPNV mRNAs can substitute for the cap function by recruiting the translation factor eIF4E and supporting translation when cap-dependent initiation is restricted by the addition of rapamycin in infected cells [119] (Figure 1, step 5). The precise eIF4E-binding site on VPg is not yet defined: The VP1 sequence contains four YXXXXLΦ motifs (where X represents any amino acid, and Φ is a hydrophobic amino acid) known to mediate eIF4E interactions with other proteins [126,127]. However, crystallographic analysis indicates these motifs are not solvent-exposed, although they lie on the face of VP1 opposite the experimentally mapped autoguanylation (RNA-attachment) site [10,84,103]. This spatial arrangement is compatible with non-overlapping surfaces for RNA linkage and eIF4E recruitment.

Moreover, segment A harbors an internal ribosome entry site (IRES) that directs translation of the polyprotein [86]. This implies that polyprotein synthesis may occur through a mechanism involving both the IRES element and the VPg protein, ensuring efficient production of structural components.

**Figure 3:**
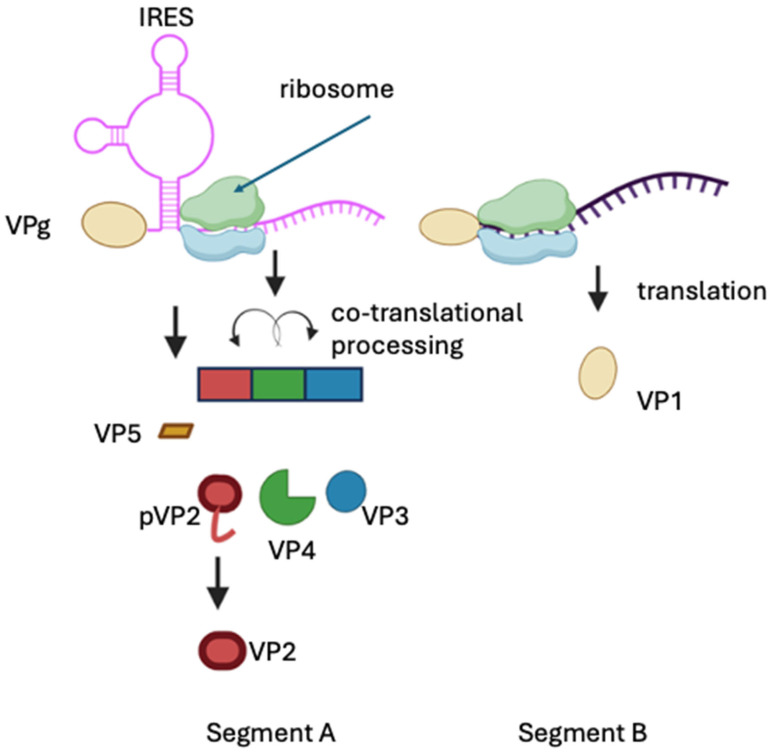
Translation of segment A mRNA occurs through distinct mechanisms. The large open reading frame encoding the viral polyprotein is translated via an internal ribosome entry site (IRES) located within the 5′ region of the segment. In contrast, the small overlapping ORF encoding VP5, positioned in the +1 reading frame relative to the polyprotein ORF near the 5′ end, is proposed to be translated in a VPg-dependent manner. The polyprotein undergoes co-translational processing by the viral protease VP4, generating pVP2 and VP3, the latter functioning as an RNA-binding protein. pVP2 is further proteolytically cleaved at residues 442, 486, and 495, yielding the mature capsid protein VP2 (amino acids 1–442) and three small peptides (p1, p2, and p3). Segment B lacks an internal ribosome entry site; therefore, translation of VP1 is proposed to be strictly VPg-dependent. Created in BioRender. Rivas, A. (2026). https://BioRender.com/0bkrr5e.

On the other hand, translation of VP5 initiates at the first in-frame AUG codon, resulting in a 5′UTR of approximately 70 nucleotides, significantly shorter than the 5′UTR of the polyprotein. The predicted folding energy of this region (−45.3 kcal mol^−1^) is less negative than the −50 kcal mol^−1^ threshold typically required to inhibit ribosomal scanning [128,129], suggesting that this leader sequence is unlikely to form a stable IRES structure. Consequently, VP5 translation is more plausibly dependent on VPg-mediated initiation. Moreover, the weak Kozak context surrounding VP5 iAUG suggests a leaky scanning mechanism for VP5 synthesis [77]. Experimental analyses of infected cells have detected VP5 concurrently with the rest of the viral proteins, although in smaller amounts [56,130], indicating distinct regulatory features governing the translation of VP5 and the polyprotein. If VP5 translation indeed relies on VPg-dependent initiation, the presence of an IRES upstream of the polyprotein ORF could impede ribosomal scanning, thereby explaining the lower abundance of VP5 relative to structural proteins. The identification of both IRES- and VPg-dependent translation in segment A mRNA suggests that IPNV employs a finely tuned translational control strategy to balance expression levels of its major proteins. Although dual translation initiation mechanisms within a single mRNA have been described for full-length human immunodeficiency virus type 1 (HIV-1) [131], there is currently no evidence of any virus that employs both VPg- and IRES-dependent initiation within the same RNA molecule or in separate transcripts (Figure 3).

mRNA-B is translated in a VPg-dependent manner and lacks an IRES [119]. VPg-dependent translation must therefore be sufficiently competitive to produce enough VP1 to remain bound to IPNV RNAs (mRNAs and gRNAs) and function as the viral polymerase [119]. On the other hand, studies using RNAs lacking covalently linked VPg in IBDV have suggested that translation requires the coordinated activity of VP1 and VP3, as it was proposed that these proteins can substitute for eIF4E to enable efficient translation of IBDV mRNAs. Moreover, assays employing bicistronic reporter constructs transfected as DNA failed to detect IRES activity in either segment A or segment B [132]. These complementary strategies illustrate how birnaviruses have evolved distinct but convergent mechanisms to ensure robust protein synthesis in the absence of canonical cap-poly(A) features.

### 3.4. Replication

In IPNV, transcription and replication are temporally separated [26,89,119]. During transcription, only minimal amounts of -RNA are detected [119], and viral proteins start to be detected earlier than genomic dsRNA. In this context, two central questions arise: first, which RNA species serves as the template for complementary -RNA synthesis? One possibility is that the VPg-linked mRNAs produced during transcription subsequently act as templates for -RNA synthesis. This leads to the second question: How can the same +RNA molecules switch from serving as templates for translation to functioning as templates for RNA replication? Current evidence suggests that VPg-linked mRNAs produced during transcription may also serve as templates for -RNA synthesis; however, direct experimental evidence supporting this mechanism in IPNV is still lacking [119] (Figure 1, step 6). The molecular signal governing this switch is unknown; one possibility is that accumulating VP1 and VP3 promote recruitment of +RNAs into replication complexes rather than ribosomes. A second, not mutually exclusive possibility is that VP1/VPg directly regulates this transition. VPg at the 5′ end of viral mRNAs interacts with eIF4E, promoting viral translation [119]. This suggests that as free VP1 accumulates, it could compete for eIF4E with translating mRNAs, thereby reducing translation and favoring engagement of VPg-linked RNAs in replication complexes. This mechanism would temporally coordinate an early protein-production phase with a subsequent replication phase focused on genome amplification.

Once recruited, viral +RNAs are copied into complementary -RNA, yielding genomic dsRNA. Early evidence came from the observation that IPNV frequently packages incomplete genomes, particularly truncated forms of segment A [133]. Subsequently, analysis of replication intermediates in infected cells revealed heterogeneous populations of RNA. At 7 hpi (the same time point at which the viral genome is detected), a full-length -RNA and various +RNAs of different lengths from the 5′ end were detected [26]. During this process, the parental positive strand can be displaced from dsRNA and either continue functioning as mRNA or serve to synthesize additional negative strands, directly contributing to genome amplification, while the -RNA serves as the replication template [11,26]. Detection of distinct RNP populations, some containing fully replicated dsRNA and others enriched in TRI, suggests that replication proceeds in parallel with nucleoprotein assembly. Indeed, provirions are detected coincident with the appearance of genomic dsRNA (Figure 1, step 7) [9].

The replication process occurs entirely in the cytoplasm. In comparison, all other dsRNA viruses replicate inside the core. For example, in reoviruses, the genome is permanently enclosed within an internal T = 2 core where transcription and replication occur; only capped mRNAs are extruded through fivefold-axis pores [112]. Classic work showed that a virion-associated replicase directs unilateral -RNA synthesis on pre-existing +RNA, producing partially ds intermediates that mature into genomic dsRNA [134]. This strategy protects viral dsRNA from recognition by innate immune sensors and subsequent degradation [135]. In birnaviruses, it has been proposed that dsRNA protection is performed by VP3, since genome replication in IBDV also appears tightly linked to encapsidation, and dsRNA remains shielded either within assembling particles or bound to VP3. The precise spatiotemporal organization of IPNV replication complexes remains poorly defined (discussed later). While IBDV uses PI3P-positive early endosomes as replication/transcription condensate sites [110], whether IPNV adopts a similar membrane-based strategy or relies solely on cytoplasmic RNP granules remains unresolved.

Host factors also influence birnavirus replication. In IBDV, VP1 activity is modulated by post-translational modifications: Ser 7 phosphorylation by CDK1–Cyclin B1 facilitates polymerase function and viral growth [136]. Arg 426 methylation by PRMT5 promotes VP1 activity [137], and interaction with host eIF4AII can inhibit VP1 and restrict replication [138]. Comparable pathways in IPNV remain unexplored, representing a key gap in understanding host control of replication dynamics.

### 3.5. Processing

The IPNV polyprotein precursor is co-translationally cleaved by VP4. VP4 also performs additional intramolecular cleavages within the C-terminal region of pVP2 at residues 442, 486, and 495, generating the mature VP2 (aa 1–442) and three small peptides (p1, p2, p3) [73] (Figure 3). Mass spectrometry studies demonstrated that these peptides remain associated with the virion, and it has been proposed that they may participate in virus entry into target cells [49]. The VP4 protease belongs to the serine/lysine-dyad protease family. Its crystal structure captured a trapped acyl-enzyme intermediate, in which the catalytic Ser 633 forms a covalent ester bond with the carbonyl of Ala 716 at the C-terminus of a neighboring VP4 molecule, directly confirming the enzyme’s self-cleavage mechanism [72]. VP4 also undergoes self-cleavage at Ala 716–Lys 717, producing a truncated form (VP4t) that has been detected both in infected cells and in purified virions; however, neither the biological reason for this cleavage nor any potential function of the resulting fragment has been described [9,56,72]. Altogether, these findings indicate that VP4 is responsible for the proteolytic processing of the viral polyprotein and for the C-terminal trimming of pVP2, thereby generating the proteolytic intermediates required for subsequent steps in virion morphogenesis.

In heterologous baculovirus expression systems, the IPNV polyprotein undergoes cis-mediated processing, resulting in the accumulation of pVP2 and VP3 but not mature VP2; moreover, addition of VP4 in trans fails to promote further processing, indicating that VP2 maturation cannot be recapitulated outside the native assembly context [56]. This observation could be due to low expression or mislocalization of VP4, the absence of host co-factors normally present in infected fish cells, or the requirement for high local concentrations of both substrate and protease within replication or assembly complexes. More recently, Villanueva, R.A, et al, 2004 [9], showed that processing of pVP2 to VP2 occurs concomitantly with provirion formation, indicating that efficient proteolysis depends on the structural and spatial organization of assembly intermediates [9]. These findings imply that optimal pVP2 processing likely occurs inside the virion (coinciding with morphogenesis) rather than being a purely soluble enzymatic event. Moreover, the persistence of peptides p1–p3 in mature virions [49] suggests that cleavage and assembly are spatially and temporally coupled, although it remains unknown whether these peptides are selectively recruited to defined sites within the capsid or incorporated stochastically during lattice assembly.

In comparison, in IBDV, pVP2 processing involves additional viral and host proteolytic events. Using mass spectrometry and N-terminal sequencing, Da Costa et al. identified four small peptides (residues 442–487, 488–494, 495–501, and 502–512) derived from the C-terminal region of pVP2, all of which remain associated with virions [139]. These findings provided the first experimental evidence that pVP2 undergoes sequential cleavages during capsid maturation (Figure 1, step 8). Later studies established the underlying mechanisms: Irigoyen et al. demonstrated that the final cleavage converting pVP2 to VP2 occurs at the Ala441–Phe442 bond and is autocatalytic, mediated by the VP2 residue Asp431. The D431N mutation abolishes this activity and prevents production of infectious virus, confirming that the reaction is essential for viral maturation [140]. Moreover, the host puromycin-sensitive aminopeptidase (PurSA) cleaves at Arg 452–Arg453 to generate the intermediate pVP2-452, which promotes the assembly of the capsids. Inhibition or silencing of PurSA disrupts morphogenesis, demonstrating the participation of a host enzyme in IBDV maturation [141].

### 3.6. Packaging

The molecular sequence of encapsidation events in IPNV remains incompletely resolved. Electron microscopy observations and biochemical analyses have identified the main structural components of the virion, but not the molecular sequence of encapsidation events. Inside the mature virion, the genome is organized as an RNP complex [15]. VP3 binds both VP1 and dsRNA [14], forming stable associations essential for genome integrity. However, there is no experimental evidence that this interaction drives or nucleates capsid assembly in IPNV. Purified IPNV virions can contain incomplete dsRNA genomes or replication intermediates (an exceptional feature among dsRNA viruses, which typically package full-length genome segments with high selectivity), suggesting that the virus can encapsidate defective replication products [26,133]. This observation implies that encapsidation is not strictly coupled to replication completion, reflecting a flexible and possibly error-tolerant assembly pathway.

In contrast with other dsRNA viruses, the birnaviral pathway is highly atypical. In all dsRNA systems, genome encapsidation involves the packaging of +RNA molecules into pre-assembled cores or proceeds cooperatively through RNA–RNA or RNA–protein complexes that serve as scaffolds for capsid growth. IPNV transcribes and replicates the RNA in the cytoplasm [142,143,144]. During infection in CHSE-214, immature particles (provirions) appear around 8 hpi [9]. Maturation likely begins soon after protein translation, with mature particles (virions) being detected from approximately 10 hpi, with concomitant shell compaction to a ~60 nm icosahedral capsid (Figure 1, step 8). In this geometry, the shell consists of 12 pentameric and 120 hexameric capsomers, each built from VP2 subunits arranged as trimers [61]. Inhibition of this proteolysis by serine-protease inhibitors such as iodoacetamide or PMSF blocks particle maturation in vitro, confirming that VP4 activity is required to acquire infectivity [9].

In comparison, the packaging process of IBDV has been characterized in greater detail. IBDV morphogenesis is organized around the multifunctional protein VP3, which simultaneously binds dsRNA, recruits VP1, and interacts with the capsid precursor pVP2 [145,146]. The 71-residue C-terminal domain of pVP2 mediates this interaction and is indispensable for the initiation of capsid assembly [146]. Once VP4 proteolytically removes this domain, pVP2 rearranges into mature VP2, closing the T = 13 shell [147,148]. Structural studies showed that VP3 forms a dimeric scaffold that coats the genome and anchors it to the inner capsid surface [145,149]. This internal network not only organizes the genome but also increases capsid rigidity, providing simultaneous scaffolding and dsRNA-binding activities. Moreover, VP3 enhances the polymerase activity of VP1, thereby coordinating replication and encapsidation in a spatially regulated manner [20]. Purified IBDV RNPs remain transcriptionally active in vitro [106], confirming that the VP1–VP3–dsRNA complex is an autonomous replication unit, a property not observed in IPNV.

On the other hand, no packaging signal has been discovered in IPNV. However, in IBDV, the stem-loop located within the 3′UTR, also conserved in IPNV, has been shown to be essential for viral replication and has been proposed to contain an encapsidation signal [87]. However, it is difficult to envision how such a loop could form within a double-stranded RNA molecule unless assisted by interactions with proteins such as VP3 or VPg.

### 3.7. Release

Most experimental evidence associates IPNV particle release with cell death processes, primarily apoptosis and necrosis (Figure 1, step 8). In relation to these mechanisms, there is still debate about whether apoptosis constitutes an indispensable preliminary step or, conversely, whether necrosis represents the predominant cytopathic event, a difference that appears to depend on the viral strain [150,151,152]. Studies with the Ab strain (E1-S) demonstrated that, in this strain, apoptosis is an early event, followed by necrosis in CHSE-214 cells. Internucleosomal DNA fragmentation was detected as early as 8 hpi, confirmed by electrophoresis and TUNEL assay, reaching 51% apoptotic nuclei by 12 hpi [151]. Moreover, electron microscopy revealed the presence of small pores in the plasma membrane (0.39–0.78 µm), a distinctive feature of IPNV-induced atypical apoptosis, which is characterized by an early loss of membrane integrity [152]. At the molecular level in the same strain, infection induces the expression of the pro-apoptotic gene Bad from 2 hpi, reaching a peak at 4 [130]. The use of inhibitors such as genistein blocks both Bad expression and apoptotic morphology. In zebrafish models, infection activates initiator caspases 8 and 9 and effector caspase 3, as well as TNF-α expression during early stages (0–6 hpi), which modulates Bad/Bid-mediated apoptosis and secondary necrosis via the RIP1/ROS pathway [153,154]. In addition, IPNV triggers ER stress–mediated apoptosis through the PKR/PERK/eIF2α–CHOP pathway, leading to Bcl-2 family downregulation [118].

In contrast, experiments with the VR299 strain showed that apoptosis occurs only marginally. Double staining assays with Annexin V (to evaluate apoptosis) and propidium iodide (to evaluate necrosis) in CHSE-214 cells showed that the apoptotic fraction never exceeded 12% of the infected population throughout the replication cycle, while necrotic cells increased steadily, reaching 75% at 15 hpi [150]. This indicates that apoptosis rarely precedes necrosis in this strain. Similar results were observed in studies with recombinant Sp serotype strains, where the percentage of apoptotic cells did not exceed 20% [77,115,116]. In this context, differences between strains suggest that IPNV modulates cell death to optimize its replication strategy: While some strains, such as E1-S, induce early apoptosis followed by necrosis, other strains delay apoptotic activation, possibly to extend cell viability and maximize viral production before the onset of lytic death.

IPNV has been proposed to manipulate cell death through VP5, which contains putative BH domains resembling those of the Bcl-2 family and has been proposed to act as an anti-apoptotic regulator. In the E1-S strain, VP5 expression was associated with delayed DNA fragmentation, maintenance of membrane integrity, and prolonged cell survival through the stabilization of Mcl-1 and modulation of viral protein expression [130]. However, the functional relevance of VP5 is highly strain-dependent: in VR299 and Sp serotypes, mutants lacking VP5 replicated efficiently both in vitro and in vivo [78], revealing that the protein is dispensable for both in vitro and in vivo replication, with no differences in kinetics or cytopathic morphology. The absence of VP5 did not increase apoptosis frequency in culture or in hepatocytes of infected Atlantic salmon, indicating that the virus can induce apoptosis independently of this protein [77]. This finding, together with the observation that several natural isolates such as Hecht or ChRtm213 possess truncated or absent VP5 open reading frames [64], strongly suggests that VP5 is a non-essential accessory factor whose anti-apoptotic activity may represent a vestigial or strain-specific trait rather than a conserved virulence determinant.

In contrast to IPNV, IBDV VP5 exhibits a clearly defined dual function: early in infection, it facilitates non-lytic egress by accumulating at the plasma membrane through its polycationic C-terminal tail that interacts with phosphoinositides, while in later stages, it promotes apoptosis via interaction with the mitochondrial voltage-dependent anion channel VDAC2 [155,156]. These mechanisms contrast sharply with those observed in IPNV, where VP5 does not alter membrane permeability [77] and its anti-apoptotic role varies between strains.

Finally, focusing solely on the apoptosis/necrosis dichotomy may overlook alternative routes of viral release. Infection with the Jasper strain (ChRtm213) actively induces autophagy in CHSE-214 cells, evidenced by the formation of double-membrane vacuoles and the conversion of LC3-I to LC3-II [157]. Unlike apoptosis, which often represents a host defense, autophagy is hijacked to favor viral replication: Its pharmacological induction with rapamycin increased viral gene expression and infectious titers, while inhibition had the opposite effect. These observations suggest that IPNV can manipulate multiple cell death pathways—apoptosis, necrosis, and autophagy—to fine-tune host cell fate and optimize the release of progeny virions [158].

## 4. Conclusions and Future Perspectives

Although IPNV has been the subject of research for more than five decades, many aspects of its biology remain poorly understood. This lack of mechanistic insight directly limits our ability to explain how the virus interacts with its host, what drives changes in virulence among emerging strains, and how IPNV continues to cause outbreaks even in fish carrying resistance QTLs. Despite significant progress in genomic and epidemiological characterization, the molecular determinants that explain these phenomena are still unknown. The first step toward overcoming these challenges is to fully understand the molecular biology of the virus and the progression of its replicative cycle. Only by defining how IPNV replicates, assembles, and interfaces with host cellular pathways will it be possible to develop targeted and effective antiviral strategies and vaccines capable of preventing infection and controlling viral persistence in aquaculture systems.

The first knowledge gap lies at the earliest stage of infection—entry and uncoating. Although receptor candidates have been identified, how their engagement triggers internalization or uncoating remains uncertain. In contrast to IBDV, IPNV does not require endosomal acidification, raising the question of what triggers membrane penetration and RNP release. The VP2 protein conserves membrane-perforating peptide sequences analogous to those that mediate endosomal escape in IBDV, although there is no experimental evidence that they are activated during IPNV entry. This phase of the viral cycle is crucial because it determines both tissue tropism and the activation of innate immune sensors.

Once inside the cytoplasm, transcription and replication define the central paradox of IPNV: What constitutes the switch from translation to the replication of the +RNAs? The discovery that IPNV mRNAs are covalently linked to VPg has challenged conventional distinctions between positive- and dsRNA viruses. Yet, the molecular “switch” that redirects these RNAs from translation to replication remains unknown. Deciphering this transition is more than a mechanistic curiosity: it would explain how IPNV achieves a delicate balance between producing enough structural proteins and amplifying its genome, while avoiding detection by the host immune system. This principle likely underlies the persistent, low-level infections typical of birnaviruses in nature.

IPNV translation itself presents several unique features. Its combination of VPg-dependent and IRES-driven initiation represents a rare hybrid strategy among RNA viruses. This duality may ensure a controlled stoichiometry between structural and enzymatic proteins, optimizing replication efficiency. However, the molecular interface between VPg and the host translation machinery remains poorly defined. VPg binds eIF4E and can support translation when cap-dependent initiation is inhibited. Viral manipulation of host stress responses (through PKR and PERK activation leading to eIF2α phosphorylation) may selectively suppress host translation while maintaining viral mRNA translation. Exploring these interactions between VPg and host translation factors could shed light on how RNA viruses adapt to stress responses and maintain replication under adverse conditions, an ability directly linked to virulence and persistence.

During the replication phase, the virus must synthesize the –RNA to generate the dsRNA genome. While the semi-conservative strand-displacement model is consistent with experimental data, the molecular initiation of -RNA synthesis has not been elucidated. The possibility that VPg-linked mRNAs directly serve as templates for –RNA synthesis remains to be tested, as does the role of cellular membranes or organelles in spatially organizing replication complexes. Whether IPNV, like IBDV, utilizes PI3P-positive endosomal membranes as replication sites is a critical open question. Such compartmentalization could explain how birnaviruses replicate in the cytoplasm while shielding dsRNA intermediates from innate immune sensors.

The subsequent stage of proteolytic processing and morphogenesis also contains several unresolved elements. VP4 orchestrates the cleavage of the polyprotein into VP2, VP3, and small peptides (p1–p3) that remain associated with the virion, yet the structural or regulatory functions of these peptides are unknown. Their persistence within mature virions suggests roles in capsid stabilization, uncoating, or genome packaging. VP4 also undergoes self-cleavage, producing a truncated VP4t of unclear function. Whether this event is autoregulatory or linked to virion maturation remains unexplored.

In parallel, the O-linked glycosylation of VP2 introduces a distinct structural variable. This modification is remarkable among viruses, representing a cytoplasmic, non-ER–mediated process. The unusual cytoplasmic glycosylation of VP2 may also contribute to virion stability and adaptation to environmental stress, representing a unique post-translational control mechanism among RNA viruses.

The assembly of genomic RNPs and their encapsidation into virions constitute another poorly defined stage. The stoichiometry and topology of VP1 and VP3 within the virion are unresolved, and structural information for the RNP complex is lacking. It is unclear whether VP3 mediates selective segment recognition or simply stabilizes any dsRNA available. The observation that IPNV can package incomplete or truncated genome segments suggests that encapsidation is not strictly sequence-specific and that the virus may tolerate replication errors, potentially promoting genetic plasticity and persistence. The conserved 3′UTR inverted repeats and predicted stem–loop motifs could serve as packaging signals, analogous to those proposed in IBDV, where disruption of 3′UTR structures abrogates infectivity but rapidly reverts. Demonstrating such structural–functional coupling in IPNV remains an important objective for future research.

In the final phase of infection, the roles of apoptosis, necrosis, and autophagy are still debated. The small nonstructural protein VP5 exemplifies this uncertainty: initially described as anti-apoptotic, its presence or absence appears to have strain-specific effects. Understanding whether VP5 truly modulates cell death or simply reflects evolutionary redundancy could illuminate how IPNV maintains persistent infections without eliminating host cells. Such knowledge is especially relevant for aquaculture, where asymptomatic carriers represent a major challenge for disease control.

In conclusion, IPNV exemplifies how a genetically simple virus can deploy highly sophisticated and still poorly understood molecular strategies to persist, adapt, and cause disease in its host. Bridging the current gaps in our understanding of IPNV entry, genome expression, replication, and morphogenesis will require integrative approaches combining virology, cell biology, structural biology, and host–virus interaction studies. Advancing from descriptive genomics toward a mechanistic dissection of the viral life cycle is essential not only to resolve long-standing biological questions but also to inform the rational design of next-generation antiviral interventions and vaccines. Such knowledge will be critical to effectively control IPNV persistence and emergence in aquaculture systems.

## Data Availability

No new data were created or analyzed in this study.

## Abbreviations

The following abbreviations are used in this manuscript:

IPNV	Infectious Pancreatic Necrosis Virus
IBDV	Infectious Bursal Disease Virus
DXV	Drosophila X Virus
MoXV	Mosquito X Virus
RBV	Rotifer Birnavirus
TV-1	Tellina Virus 1
VR299	VR299 strain
E1-S	E1-S strain
Sp	Spajarup strain
WB	West Buxton strain
DM	Dry Mills strain
DRT	DRT strain
Ja	Jasper strain
C1	Canada-1 strain
C2	Canada-2 strain
C3	Canada-3 strain
Ab	Abild strain
Te	Tellina strain
He	Hecht strain
VPg	Viral Protein Genome-linked
RNA	Ribonucleic Acid
DNA	Deoxyribonucleic Acid
dsRNA	Double-Stranded RNA
RNP	Ribonucleoprotein Complex
RdRp	RNA-Dependent RNA Polymerase
ORF	Open Reading Frame
UTR	Untranslated Region
IRES	Internal Ribosome Entry Site
TRI	Transcription Intermediate
ASK cells	Atlantic Salmon Kidney cells
CHSE-214	Chinook Salmon Embryo cell line
SHK-1	Salmon Head Kidney cell line
Cdh1-1	Cadherin-1-1
Myh9	Non-Muscle Myosin Heavy Chain 9
IFN	Interferon
Mx	Myxovirus Resistance Protein
PKR	Protein Kinase R
TNF-α	Tumor Necrosis Factor Alpha
UPR	Unfolded Protein Response
ER	Endoplasmic Reticulum
PERK	PKR-Like Endoplasmic Reticulum Kinase
eIF2α	Eukaryotic Initiation Factor 2 Alpha
eIF4E	Eukaryotic Initiation Factor 4E
CDK1	Cyclin-Dependent Kinase 1
Bcl-2	B-Cell Lymphoma 2
Bad	Bcl-2-Associated Agonist of Cell Death
Bid	BH3 Interacting Domain Death Agonist
RIP1	Receptor-Interacting Protein Kinase 1
TUNEL	Terminal Deoxynucleotidyl Transferase dUTP Nick End Labeling
VDAC2	Voltage-Dependent Anion Channel 2
NTP	Nucleoside Triphosphate
GMP	Guanosine Monophosphate
PMSF	Phenylmethylsulfonyl Fluoride
PI3P	Phosphatidylinositol-3-Phosphate
O-GlcNAc	O-Linked N-Acetylglucosamine
H^+^-ATPase	Proton-Translocating ATPase
PurSA	Puromycin-Sensitive Aminopeptidase
QTL	Quantitative Trait Locus
